# Ablation of Tumor Necrosis Factor Alpha Receptor 1 Signaling Blunts Steatohepatitis in Peroxisome Proliferator Activated Receptor α-Deficient Mice

**DOI:** 10.18103/mra.v10i9.3082

**Published:** 2022-09-20

**Authors:** Ian N. Hines, Jamie Milton, Michael Kremer, Michael D. Wheeler

**Affiliations:** 1 Department of Nutrition Science, East Carolina University, North Carolina, USA; 2 Department of General and Visceral Surgery, Hospital of Aarau, Aarau, Switzerland

**Keywords:** liver, inflammation, immune cell, cytokine

## Abstract

Tumor necrosis factor -alpha (TNFα) is strongly associated with fatty liver disease (i.e, hepatosteatosis). Cytokine production has been thought of as a consequence of hepatic lipid accumulation which becomes a critical factor in the development of chronic liver pathologies as well as insulin resistance. The purpose of this study was to test the hypothesis that TNFα directly regulates lipid metabolism in liver in the mutant peroxisome-proliferator activated receptor-alpha (PPARα^−/−^) mouse model with robust hepatic lipid accumulation. At 10 weeks of age, TNFα and TNF receptor 1 expression are increased in livers of PPARα^−/−^ mice compared to wild type. PPARα^−/−^ mice were then crossed with mice lacking the receptor for TNFα receptor 1 (TNFR1^−/−^). Wild type, PPARα^−/−^, TNFR1^−/−^, PPARα^−/−^ x TNFR1^−/−^ mice were housed on ad-libitum standard chow diet for up to 40 weeks. Increases in hepatic lipid and liver injury and metabolic disruption associated with PPARα ablation were largely blunted when PPARα^−/−^ mice were crossed with TNFR1^−/−^ mice. These data support the hypothesis that TNFR1 signaling is critical for accumulation of lipid in liver. Therapies that reduce pro-inflammatory responses, namely TNFα, could have important clinical implications to reduce hepatosteatosis and progression of severe liver disease.

## INTRODUCTION

Non-alcoholic fatty liver disease (NAFLD) is a rapidly growing cause of liver damage, dysfunction, and failure in westernized countries ^1^. Estimates within the United States alone predict greater than 22% of the population suffers from some form of this spectrum disorder. Indeed, hepatocellular steatosis, or the accumulation of lipid within hepatocytes, was once thought to be a benign pathology with little effect on cell or tissue function ^2^. Indeed, NAFLD is characterized as a progressive pathology beginning with simple steatosis and evolving, over time, to steatohepatitis (NASH) with significant inflammatory cell infiltrate and hepatocellular damage, and ultimately in a smaller percentage of patients (~20% of patients with NASH) to tissue scarring or fibrosis and cirrhosis ^3^. Key experimental studies have defined a number of factors which contribute to NAFLD disease induction and evolution ^4^. From these, it is clear that early activation of hepatic macrophages, the Kupffer cell, by gut-derived factors including endotoxin promote Tlr4 dependent expression of a variety of inflammatory cytokines and promote inflammatory cell infiltrate, hepatocellular mitochondrial dysfunction, and lipid metabolism disruption ^5 6 7^. Indeed, Miura and others, using the choline deficient diet model of NAFLD, demonstrate the profound importance of macrophages and their recruitment through inflammatory chemokine receptors, specifically CCR2, to promote lipid accumulation and progression towards fibrogenesis ^8^. Moreover, loss of Tlr4 was shown to limit lipid accumulation in a similar model of fatty liver in mice ^5^. Together, it is clear that inflammation and inflammatory cytokines derived from or initiated by resident immune cells propagate hepatocellular injury and disruption of critical metabolic programs leading to lipid accumulation.

While it is still not fully understood how inflammatory cytokines impact hepatic metabolism or likewise, how altered hepatic metabolism affects inflammatory responses, a growing body of experimental data would implicate certain pro-inflammatory cytokines as potential initiators and / or propagators of non-alcoholic steatohepatitis (NASH) ^9^. Increased lipid accumulation itself promotes hepatocyte dysfunction, hepatocellular oxidant stress, and activation of hepatic innate immunity ^10^. Moreover, lipid accumulation depletes antioxidants and increases oxidative stress through lipid peroxidation, both of which may promote DNA damage and hepatic carcinogenesis ^2,10,11^. One potential hypothesis is the lipid accumulation within liver, as with the case of high-fat diet, hyper-caloric diet or ethanol containing diet, activates inflammatory cascades resulting in cytokine release that propagates further inflammatory cell recruitment and hepatocellular damage.

Tumor necrosis factor alpha (TNFa) is a key cytokine produced by a variety of inflammatory cells including macrophages as well as by hepatocytes during periods of stress ^12 13^. Signaling primarily through two TNF receptors (TNFR1 and TNFR2), TNFa is associated with a variety of cellular responses from cell proliferation and differentiation to apoptosis and regulation of the immune response ^14^. Within the liver, TNFa is well appreciated for its ability to promote inflammation through propagation of macrophage function while its production promotes the regenerative response following hepatectomy ^14 15 16^. Intriguingly, TNFa is also associated with mitochondrial dysfunction ^7^. Recent studies have highlighted its capacity to decrease mitochondrial respiration in aging platelets as well as in neuronal cells *in vitro* ^17 18^. Similarly, very early work with isolated hepatocytes showed reduced mitochondrial respiration in response to TNFa exposure *in vitro*, data which was later extended showing a direct nuclear factor kappa B (NFkB) mediated, reactive oxygen species dependent uncoupling of complexes I and III ^19 20^. Likewise, TNFα also remains a clear link between innate immunity and obesity and metabolism, particularly within the liver ^21^. To this point, mice lacking the TNF receptor 1 show resistance to diet-induced insulin resistance ^22^. In summary, TNFa plays an important role in hepatic inflammatory responses and contributes to altered metabolic function both within the liver as well as peripherally.

Mice lacking the gene for peroxisome proliferator-activated receptor a (PPARα^−/−^) develop robust fatty liver disease within 20 weeks of age and severe liver disease within 40–60 weeks of age ^23^. Mechanistically, PPARα is a transcriptional regulator of acyl CoA oxidase and thus is a critical regulator of hepatic fatty acid oxidation ^24^. Since the accumulation of lipid is related to an impairment in lipid oxidation in PPARα^−/−^ mice, these mutant mice are a useful model to investigate the effects of lipid accumulation in liver in contrast to dietary models where nutrient composition, intake, or the bio-active effect of nutrients on gut microbiota, metabolism or immune response play a role. Importantly, PPARα^−/−^ mice also exhibit an increase in TNFα expression in liver and this observation may suggest that TNFα pathways may be activated in response to lipid accumulation or possible causal in the lipid accumulation that leads to subsequent liver injury.

### Objective:

To better understand the role of TNF receptor dependent signaling in the development and progression of fatty liver disease, PPARα-deficient mice were crossed with mice lacking TNFR1 deficient mice. Using this well-defined genetic model of dysregulated lipid metabolism and consequent fatty liver development which is independent of dietary variables, this study will determine the influence of TNF signaling directly on metabolic responses in this paradigm within the murine liver. Information gathered here will better define the factors responsible for fatty liver development and provide possible therapeutic targets to treat or prevent NAFLD.

## DETAILED METHODS

### Animals and treatment.

Male C57Bl/6J wild type mice or TNFα receptor 1-deficient (TNFR1^−/−^; C57Bl/6- *Tnfrsf1a*^*tm1Imx*^*/J*) mice were purchased from Jackson laboratories (Bar Harbor, ME) while peroxisome proliferator activated receptor alpha-deficient mice (PPARα^−/−^, B6.129S4-*Ppara*^*tm1Gonz*^ N12) on a C57Bl/6 background were obtained from Taconic (Hudson, NY). The PPARα and TNFR1 double (DKO) deficient mice were generated by cross-breeding in house. The genotypes of all animals were verified by standard PCR procedures using primer sequences from the suppliers. Each strain was maintained through established breeding protocols and kept within AALAC approved facilities and guidelines. The procedures for the care and treatment of mice were followed according to those set by East Carolina University Institutional Animal Care and Use Committee guidelines. Male mice of each genotype were then fed a standard lab diet (Prolab RMH 3000, LabDiet. St. Louis, MO) for up to 40 weeks of age. Following feeding, the mice were sacrificed and serum and tissue collected for further analysis of routine parameters of liver injury and lipid accumulation.

### Live animal monitoring.

NMR-MRI (EchoMRI, Houston TX) analyses were performed for body composition measurements of fat, lean, free water and total water masses in live mice.

### Body mass index.

Body weight was measured for each mouse prior to euthanasia. Crown-rump length defined as the distance between the crown of the skull and a point located in the middle of a line between the two caput femoris was measured. The BMI was then calculated as the body weight (g)/[crown-rump length (mm)]^2^.

### Measurements of Serum Parameters.

Serum levels of alanine aminotransferase and triglycerides were measured by spectrophotometric analysis (Sigma-Aldrich, St. Louis MO). Clinical chemistry was performed by the University of North Carolina Clinical Chemistry laboratory. Serum glucose levels were determined using a glucose analyzer (Beckman, Fullerton, California) while insulin, leptin, and adiponectin were measured via radioimmunoassay as previously described ^25^.

### Histopathology and Immunohistochemistry.

Tissue was fixed in 4% phosphate buffered formalin for 24 hours and subsequently embedded in paraffin. Tissue sections were prepared (7μm thick) and subjected to routine hematoxylin and eosin staining.

### Real time Reverse Transcriptase Polymerase Chain Reaction.

Total RNA from liver was isolated using the Trizol reagent (Gibco/ ThermoFisher Scientific, Grand Island NY) according to the manufacturer’s recommendations. Total RNA (1μg) was used to synthesize cDNA using the High-Capacity cDNA Reverse Transcription kit from Applied Biosystems. For quantification of message expression, cDNA was amplified using specific primer sequences for murine TNFα (F- 5’-AGCCCACGTAGCAAACCACCAA-3’; R- 5’-ACACCCATTCCCTTCACAGAGCAAT-3’), TNF receptor 1 (F- 5- ’-GCCCGAAGTCTACTCCATCATTTG-3’; R- 5’GGCTGGGGAGGGGGCTGGAGTTAG-3’), and b actin (F - 5’-AGGTGTGCACCTTTTATTGGTCTCAA-3’; R - 5’-TGTAGTAAGGTTTGGTCTCCCT-3’) in the presence of Taq polymerase and Sybr Green using a kit from Applied Biosystems/ ThermoFisher Scientific (Grand Island NY) using a standard PCR protocol (95°C for 10s, 57°C for 15s, and 72°C for 20s, total of 40 cycles. β-actin message expression was used as the house keeping gene and for quantification of relative expression levels using the comparative cT method of quantification.

### Statistical Analysis

Data are presented as mean ± standard error of the mean (SEM) of 3 or more animals per group. Data were analyzed using non-parametric Student’s t-Test where significance was set at p<0.05.

## RESULTS

**Figure 1.**
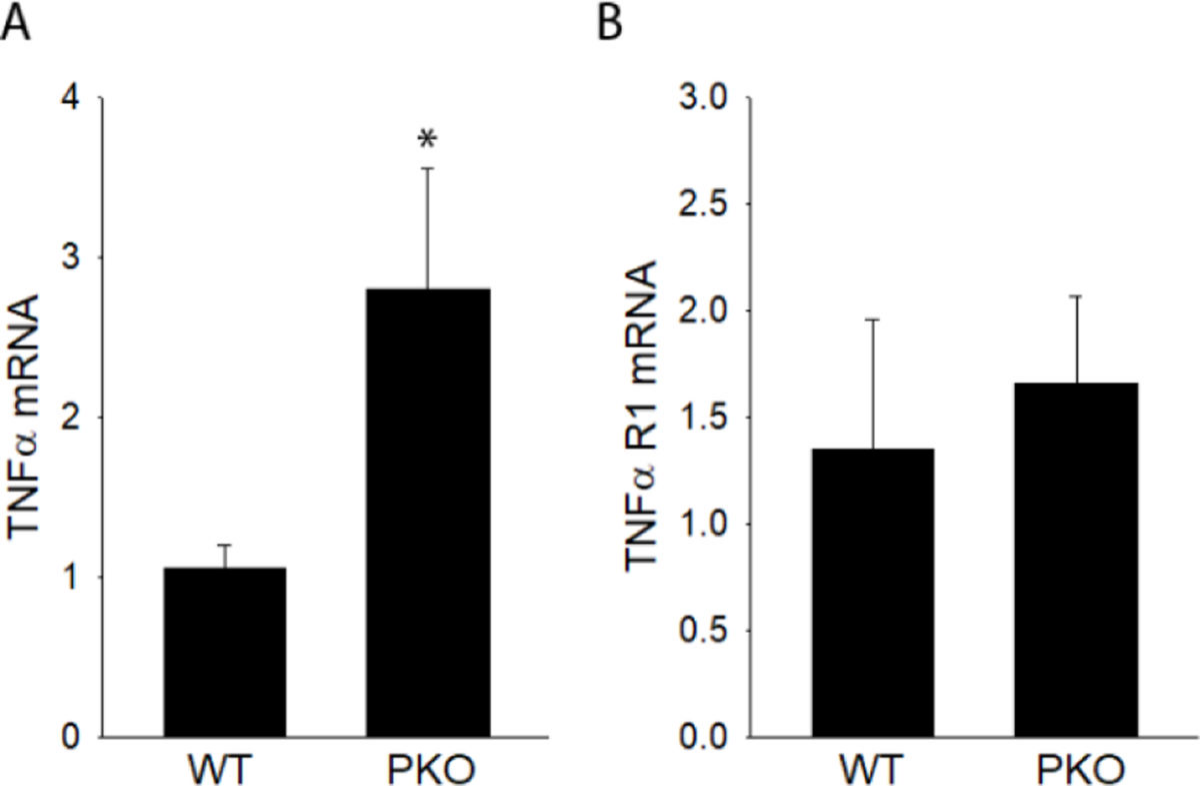
TNFα and TNFR1 expression in PPARα−/− mice. Liver mRNA levels of TNFα and TNF receptor 1 was measured by quantitative PCR in wildtype and PPARα−/− (PKO) mice at 10 weeks of age. Data are representative of 3–6 animals per group and are expressed as mean SEM. t-test was performed. *, p<0.05, compared to wild type mice.

It is documented elsewhere that ablation of PPARα results in age-dependent lipid accumulation within liver ^26^. Here, it is demonstrated that PPARα^−/−^ mice (PKO) also exhibit increased mRNA levels of TNFα at 10 weeks of age. Compared to wild type mice, the level of mRNA for TNFα was increased 2.8-fold in livers of PPARα^−/−^ mice at this early timepoint (Figure 1). The levels of mRNA for TNFR1p55 in liver were not, however, significantly elevated in PKO mice when compared to their wild type controls at 10 weeks of age. The increase in TNFα expression in liver precedes the known accumulation of hepatic lipid associated with the ablation of PPARα. These findings support the hypothesis that the TNFα pathway plays a causal role in the liver pathology associated with PPARα ablation.

**Figure 2.**
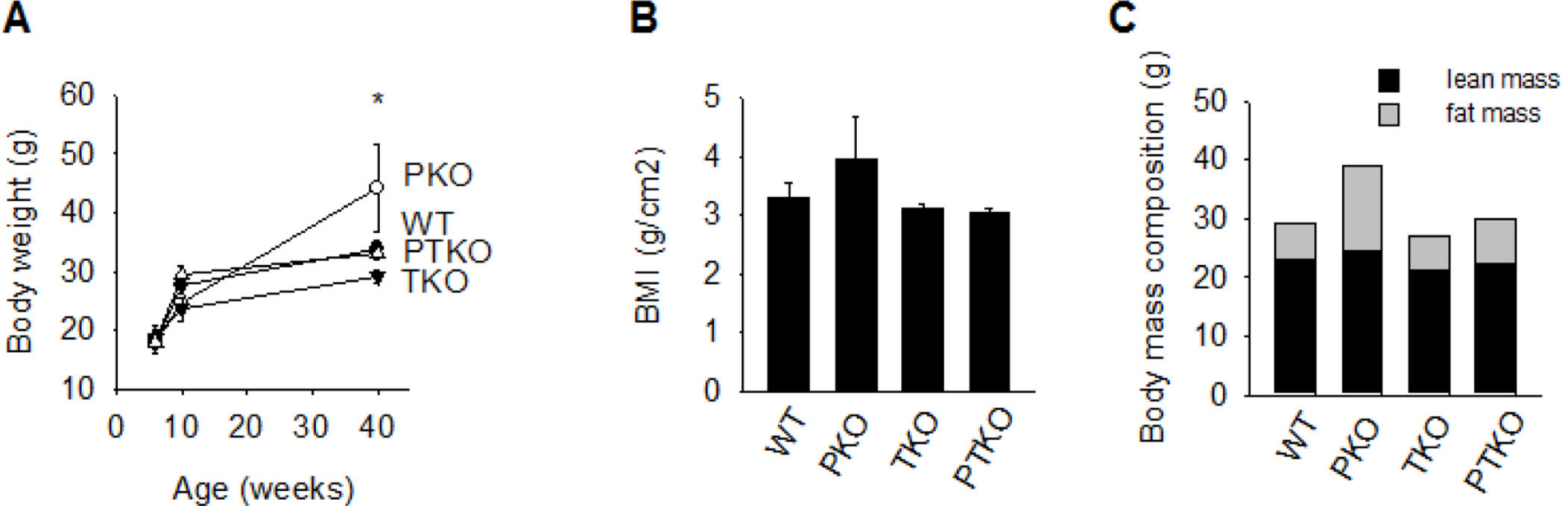
Loss of TNFα receptor blunts adiposity in PPARα-deficient mice. **A.** Body weight changes at 6, 10 and 40 weeks of age in wild type (WT), PPARa deficient (PKO), TNFaR1 deficient (TKO), and PPARa and TNFaR1 double deficient (PTKO) mice. **B**, Body mass index was emperically measured in 40 week old animals. **C**, NMR-MRI was used to determine fat and lean tissue mass in 40 week old mice. Data are representative of 3–6 animals per group and are expressed as mean ± SEM. *, p<0.05, compared to wild type mice at similar age; Two-way ANOVA with Bonferoni post-hoc analysis was performed.

To address the role of TNFα in a genetic model of hepatic steatosis, TNFR1^−/−^ mice (TKO) were crossed with mice lacking peroxisome proliferator activated receptor alpha (PKO) to generate double mutant PPARα^−/−^ TNFR1^−/−^ mice (PTKO). Mice were housed in 12-hour light-dark cycles and were provided standard chow diet ad libitum. A significant increase in body weight was observed in PKO mice compared to that of wild type (WT) mice (Figure 2) at 40 weeks of age. The weight gain in both TKO and PTKO mice was not significantly different from WT mice. Body fat composition of each strain was determined by magnetic resonance. Lean muscle mass was not significantly different among strains. The fat mass percentage of WT mice was 18.8±3.0% while body fat content in PKO mice at 40 weeks of age was 24.6±1.8%. Importantly, the body fat percentage in TKO and PTKO mice at similar ages was 13.0 and 13.8%, respectively (Figure 2). The changes in body fat composition correlated with BMI, which was determined at sacrifice. The increase in BMI observed in PKO compared to WT mice was significantly blunted in PTKO mice.

**Figure 3:**
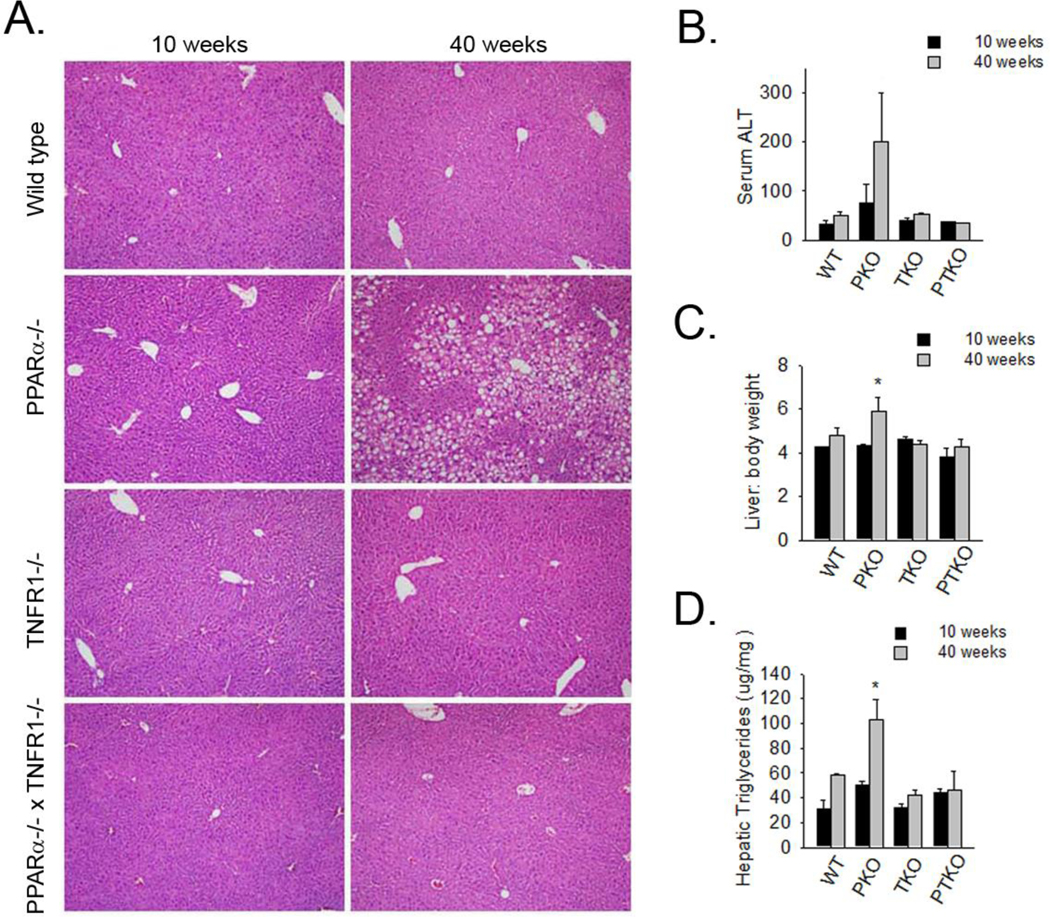
Loss of TNF receptor blunts liver injury and hepatic fat accumulation in PPARa-deficient mice. Wild type (WT), PPARa deficient (PKO), TNFaR1 deficient (TKO), and PPARa and TNFaR1 double deficient (PTKO) mice were sacrificed at 10 weeks and 40 weeks of age. **A.** Representative liver histology (H&E) presented at 10X magnification. **B.** Serum alanine aminotransferase levels at 10 and 40 weeks of age. **C.** Liver weight to body weight ratios at 10 and 40 weeks of age. **D.** Hepatic tissue triglyceride levels at 10 and 40 weeks of age. Data representative of 3 to 6 mice per group and are expressed as mean ± SEM. *p<0.05 vs. similar aged wild type controls.

Loss of PPARa is well appreciated to result in hepatosteatosis. Livers from PKO mice at 10 weeks of age had evidence of very mild fat accumulation where wild type had exhibited normal histology, but at 40 weeks of age, PKO mice exhibited severe fat accumulation and mild inflammation which was not observed in similarly aged WT controls. Hepatosteatosis was not observed after 10 weeks or 40 weeks in TKO mice. Importantly, hepatosteatosis was absent in PTKO mice after 10 and 40 weeks (Figure 3). Serum ALT levels were measured at 10 and 40 weeks of age in all groups. Loss of PPARa led to an increased serum ALT levels at 40 weeks of age when compared to similarly aged wild type mice. This increase was blunted in PTKO mice. Liver weight was measured at euthanasia and liver to body weight ratio was determined. Liver to body weight ratio, indicative of hepatosteatosis, was significantly increased in PKO compared to WT mice at 40 weeks of age (Figure 3). Like body weight and fat mass, the liver to body weight ratio was similar to WT in both TKO and PTKO mice.

Liver triglyceride levels were significantly elevated (approximately 2-fold) at 40 weeks in PKO mice, compared to the level in WT mice at similar time points. In TKO mice, liver triglyceride levels were similar to that of WT animals at both 10 and 40 weeks. In PTKO mice, liver triglyceride levels were only mildly elevated after 40 weeks. Importantly, the liver triglyceride levels were significantly blunted in PTKO mice compared to PKO mice (Figure 3). These data support the hypothesis that fatty liver due to loss of PPARα is dependent upon TNFR1 receptor expression.

**Figure 4.**
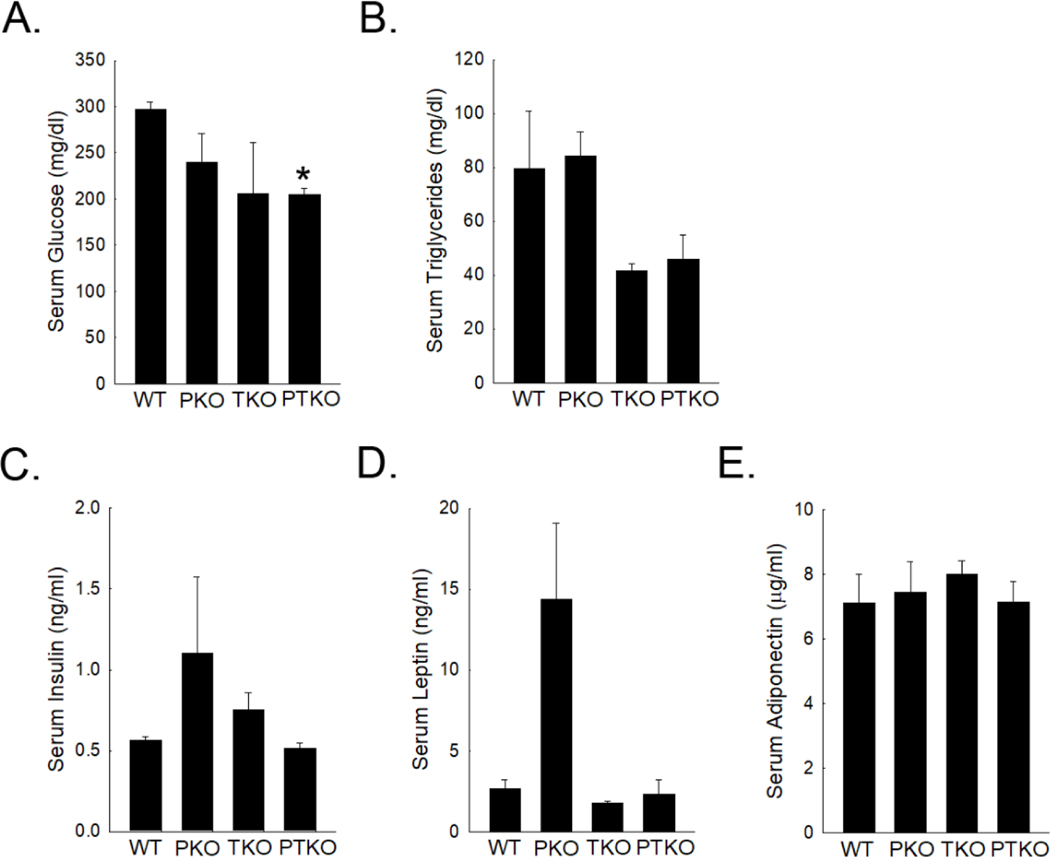
Effect of loss of TNFa on the system metabolic response in PPARa-deficient mice. Serum levels of glucose (A), triglycerides (B), insulin (C), leptin (D), and adiponectin (E) were measured in wild type (WT), PPARa deficient (PKO), TNFaR1 deficient (TKO), and PPARa and TNFaR1 double deficient (PTKO) mice at 40 weeks of age. Data presented as mean ± SEM for 3 animals per group.

To further understand the impact of TNFa on the systemic metabolic response in PKO mice, serum levels of glucose, triglycerides, insulin, leptin, and adiponectin were measured (Figure 4). Following 40 weeks of chow feeding, wild type mice had serum glucose levels of 297.33 ± 6.96 mg/dL. Interestingly, loss of PPARa was associated with a reduction, although not significant, in blood glucose levels when compared to wild type controls. Absence of TNFaR1 was also associated with a reduction, although not significant, in blood glucose levels when compared to controls. Absence of both TNFaR1 and PPARa showed the most consistent reduction in blood glucose levels when compared to similarly aged wild type mice.

Serum triglycerides were also examined. Following 40 weeks of chow feeding, wild type mice presented with serum TGs at 79.66 ± 21.16 mg/dL. Loss of PPARa did not alter serum triglyceride levels at this age when compared to wild type controls (84.33 ± 8.74 mg/dL). In the absence of TNFaR1, serum levels of triglycerides were significantly reduced compared to wild type controls (41.66 ± 2.14 mg/dL). Importantly, when TNFaR1 was absent in PPARa deficient mice, serum triglycerides were also significantly reduced compared to wild type controls and PPARa deficient mice.

Selected metabolic hormone levels were also measured. Following 40 weeks of chow diet feeding, insulin levels were mildly elevated in PKO mice when compared to similarly aged wild type mice. However, when TNFa receptor was absent in PKO mice, serum insulin levels were consistent with wild type control levels. Loss of TNFa receptor alone did not alter insulin levels after 40 weeks of chow feeding when compared to wild type controls (Figure 4).

Evaluation of serum leptin levels revealed a large increase in PKO mice when compared to wild type mice at 40 weeks of age. This increase was completely abrogated in PTKO mice suggesting a role for TNFa signaling in this increase. Loss of TNFa receptor alone did not alter serum leptin levels when compared to similarly aged wild type controls. Finally, serum adiponectin levels were measured and no differences were noted in the three mutants when compared to similarly aged wild type controls (Figure 4).

## DISCUSSION

A number of pro-inflammatory cytokines and chemokines including TNFα are up-regulated in fatty livers ^27^. Likewise, innate immune responses activated within fatty livers have great potential for amplification ^28 29 5 30^. Once cytokine production is initiated, these pro-inflammatory cytokines propel the progression from steatosis to steatohepatitis. The evidence in favor of the role of TNFα in fatty liver disease is overwhelming, although direct evidence from animal models has been mixed ^31 32 33 34 35^. Moreover, the mechanism by which TNFα contributes to hepatic lipid metabolism is a gap in our understanding. TNFα is a potent inflammatory mediator derived from a variety of cell types which interacts with a wide range of signaling pathway. Importantly, we have demonstrated here and previously in an ethanol-diet model of steatosis/steatohepatitis that TNFα is crucial for the accumulation of lipid in the liver ^15^. Here, we used the TNFR1−/− deficient mice to determine its role in the general mechanisms of fatty liver disease in the genetically obese PPARα−/− mice. Importantly, these data suggest that hepatic inflammation may precede and promote the accumulation of lipid, affirming the notion that cytokines drive and potentiate hepatic metabolic dysfunction as well as the pro-inflammatory cascade.

There is some experimental evidence that TNFα is increased in response to the accumulation of lipid as well as a growing body of evidence that TNFa is also a regulator of hepatic lipid metabolism. Diehl and others demonstrated decreased lipid accumulation in TNFα-deficient mice fed a high calorie diet ^32^. This was an important finding since there is much evidence that high fat diet- similar to an ethanol containing diet- causes an increase in TNFα production in liver. Much work has also investigated c-jun N-terminal kinase (JNK), a primary downstream target of TNFR1 in hepatocytes, as a regulator of hepatic lipid accumulation ^36 37^. Indeed, JNK null mice were resistant to both methionine-choline deficient diet induced hepatosteatosis and high-fat diet induced fatty liver and secondary tissue injury and hepatocellular apoptosis resulting from this lipid overaccumulation ^36^.

A central question remains whether TNFα is a consequence of lipid accumulation that follows metabolic derangement for example due to ethanol, high fat or hyper-caloric diet or whether TNF is a driver of metabolic changes that result in fat accumulation and subsequent liver inflammation and injury. In either case, the hypothesis is that TNFα through TNFR1 pathways exacerbates liver inflammation and blunts lipid metabolic pathways, which further perpetuates the development of liver injury. The notion of a “feed forward” cycle involving lipid metabolism and pro-inflammatory cytokines is not novel. Fiengold reported that TNF suppressed lipid metabolism including an increase in serum triglyceride levels and a decrease in hepatic fatty acid oxidation, in bile acid synthesis, and in high-density lipoprotein levels ^21^. These effects of TNFα were through the suppressed expression of nuclear hormone receptors retinoid X receptor alpha (RXRa), PPARa, PPARg, and liver X receptor alpha (LXRa), as well as coactivators peroxisome proliferator-activated receptor gamma co-activator 1 alpha (PGC-1a) and PGC-1b ^21^. This observation as well as others led to the notion of a “two-hit” model of liver injury, where metabolic alterations were coupled with the pro-inflammatory responses to propagate a futile and deleterious cycle of liver injury ultimately leading to irreversible pathology.

Studies of the effects and interactions of PPARa deficiency and TNFa in the regulation of systemic metabolic were less conclusive. Consistent with previous reports, loss of PPARa led to a reduction, albeit not significant, in fasting glucose levels, a response which was furthered by the loss of TNFa signaling in these mice ^23^. This correlated with increased levels of insulin secretion in these mice when compared to wild type controls. The concomitant loss of TNFa and PPARa returned serum insulin levels to that seen in wild type controls. These findings are somewhat consistent with previous studies which demonstrated a role for TNFa in the development of insulin insensitivity ^22^. The differences likely lie within the models utilized, where high fat, high calorie diets likely magnify the baseline inflammatory response increasing the influence of factors such as TNFa and others in the metabolic processes. Similarly, loss of PPARa did not significantly alter serum triglyceride levels when compared to wild type controls. This is also consistent with previous reports showing no significant alterations in serum triglyceride levels in PPARa deficient mice on a standard chow diet ^38^. Importantly, our data again support a role for TNF signaling directly in the regulation of systemic metabolic responses. Absence of TNFa alone correlated with a significant reduction in serum TGs when compared to wild type controls. Moreover, this effect was consistent in PPARa deficient mice where PTKO mice TG levels were significantly reduced when compared to wild type mice. Early studies correlated increased TNFa levels with increased body mass index, fasting glucose levels and circulating triglycerides ^12^. Importantly, our data suggest that alterations in hepatic metabolic function and lipid accumulation does not, in the current model system, correlate with systemic alterations in lipid homeostasis, that rather, inflammatory factors including TNFa more likely influence these processes.

One interesting feature of our data is that ablation of PPARa, a regulator of hepatic lipid peroxidation, caused a large increase in serum leptin levels when compared to wild type controls, a response which was completely abrogated by deletion of TNFaR1 in these same mice. Previous studies have linked PPARa to leptin production whereby activation with exogenous ligand, gemfibrozil, decreased leptin secretion in diet-induced obese rats ^33^. Leptin is well appreciated for its influence on energy balance and a multitude of secondary effects which alter a variety of physiological processes ^29^. Specifically and intriguingly, leptin has also been associated with the induction of pro-inflammatory cytokines by a variety of cells including macrophages. The initiator of the inflammatory response, particularly in this relatively simple model, is proposed to be lipid accumulation itself however secondary factors including leptin may promote or amplify this inflammatory cascade. Likewise, local TNF production at sites of leptin production, likely the white adipose tissue, appear to initiate this inflammo-endocrine cascade. Further study is needed to define the importance of leptin production in this genetic-induced obesity model.

## Conclusion

These data highlight the importance of TNF receptor signaling in PPARα-deficient mice to facilitate lipid accumulation. TNFα, as well as other downstream pro-inflammatory cytokines, are increased as a result of PPARα ablation in mice. This effect is also observed in diet-induced hepatic steatosis. Data presented here supports the hypothesis that TNFα through its TNF receptors is a critical factor in the development of fatty liver, suggesting that TNF and the pro-inflammatory response are not merely a consequence of lipid accumulation but a major driver of the changes in lipid metabolism leading to the accumulation of lipid in liver. These findings have important implications for the role of TNF and pro-inflammatory cytokines in diet-induced fatty liver disease, not just in genetic models of steatohepatitis. Therapeutic or perhaps nutritional strategies to reduce the pro-inflammatory response represent potential early interventions for non-alcoholic fatty liver disease.
